# Predictive ability of the start back tool: an ancillary analysis of a low back pain trial from Danish general practice

**DOI:** 10.1186/s12891-017-1727-6

**Published:** 2017-08-23

**Authors:** Allan Riis, Michael Skovdal Rathleff, Cathrine Elgaard Jensen, Martin Bach Jensen

**Affiliations:** 1 Research Unit for General Practice in Aalborg, Department of Clinical Medicine, Aalborg University, Fyrkildevej 7, 1. sal, lejl. 3, 9220 Aalborg Øst, Denmark; 2 Danish Center for Healthcare Improvements, Aalborg University, Fibigerstræde 11, 9220 Aalborg Øst, Denmark

**Keywords:** General practice, Adult, Low back pain, Patient outcomes assessment, Prognosis

## Abstract

**Background:**

Low back pain (LBP) is a common cause of contact with the primary healthcare sector. In some patients, symptoms quickly resolve, but others develop long-lasting pain and disability. To improve the care pathway for patients with LBP, the STarT Back Tool (STarT) questionnaire has been developed. It helps initial decision-making by subgrouping patients on the basis of their prognosis and helps to target treatment according to prognosis. An assumption behind the use of STarT is the ability to predict functional improvement. This assumption has never been tested in a population that consists exclusively of patients enrolled when consulting a Danish general practitioner for LBP. The aim of this study was to investigate STarT’s ability to predict a 30% improvement in the Roland Morris Disability Questionnaire (RMDQ) score.

**Methods:**

This was an ancillary analysis using data from a Danish guideline implementation study (registered at ClinicalTrials.gov NCT01699256). An inclusion criterion was age 18 to 65 years of age. Exclusion criteria were pregnancy, fractures, and signs of underlying pathology. Patient-reported STarT score and the Roland Morris Disability Questionnaire were administered at baseline and again after 4, 8, and 52 weeks.

**Results:**

Between January 2013 and July 2014, 475 patients from the original trial participated with questionnaires. From this subpopulation, 441 (92.8%) patients provided information regarding STarT. Baseline and eight-week RMDQ data were available for 304 (64.0%) patients. After 8 weeks, 61 (65.6%) in the low-risk group, 67 (54.9%) in the medium-risk group, and 33 (37.1%) in the high-risk group had achieved a 30% improvement in the RMDQ score. After 8 weeks, high-risk patients were at 61% (95% CI: 20–125%, *P* < 0.001) higher risk of not achieving a 30% improvement in the RMDQ score compared with patients in either the low-risk group or the medium-risk group.

**Conclusion:**

STarT was predictive for functional improvement in patients from general practice with LBP.

**Trial registration:**

ClinicalTrials.gov NCT01699256, Nov 29, 2016 (registered retrospectively).

## Background

The Global Burden of Disease study showed that low back pain (LBP) is very common, with an estimated point-prevalence of 9.4% and, therefore, a leading contributor to disability worldwide [1]. Most episodes of LBP only last a few days, but many patients with LBP experience recurrent symptoms, and up to 45% of patients complaining of LBP who consult primary care physicians will have LBP after 1 year [2, 3]. The underlying causes of LBP are often unknown but are in many cases multifactorial, including both biological [4] and psychosocial factors that may be important for pain and recovery [5–7]. A multitude of different treatments exist, including general information on LBP, general exercises to improve the patients’ overall physical condition, specific strengths or flexibility exercises targeted at a specific physical problem, treatments aimed at work or ergonomic-related issues, personal problems, problems with family and social life, manual therapy, massage, yoga, and cognitive behavioural therapy [8–10]. Furthermore, treatments can be delivered to individuals or to groups; treatments can be supervised and performed in the healthcare setting or instructed/agreed upon to be performed at patients’ homes, public places, or at sport clubs. Hence, as the reason underlying LBP is often multifactorial, and the care of patients is complex; methods to support targeted treatment can avoid the treatment of patient characteristics unrelated to the patients’ pain [11]. Therefore, tools that are able to guide initial decision-making and that can improve care are needed. Subgrouping patients into risk strata by the STarT Back Tool (STarT) has been suggested to target treatment to modifiable factors that are causally related to outcome among sub-groups of patients presenting with LBP in primary care [12].

### The STarT back tool

STarT integrates biological, psychological, and social factors and includes nine questions that are used to subgroup patients into a low-, medium- or high-risk subgroup according to the risk of persistent disabling pain [13]. For each subgroup, the STarT follows a set of recommendations for treatment. Patients in the low-risk group are recommended to receive information on LBP and advice to stay as physically active as possible and to continue daily activities. Supplementary to information and advice, GPs are expected to recommend standardized treatment focusing on addressing physical symptoms and function to patients in the medium-risk group. In addition, healthcare professionals are expected to pay special attention to cognitive behaviour to address psychosocial obstacles to recovery for patients in the high-risk group [14]. The STarT has been found to be effective in predicting functional outcomes and has also been found to be effective when applied in two large studies in UK settings [15, 16]. Currently, stratification by the STarT is recommended in the newly published NICE guidelines [10].

### Predictive ability of the STarT back tool

Numerous studies performed in different healthcare settings have tested the predictive ability of the STarT. The findings from these studies are inconclusive, hampering widespread use across different healthcare settings [17–24]. In a recent guideline implementation trial for patients with LBP, a subgroup of patients completed a range of questionnaires, including the STarT at baseline and the Roland-Morris Disability Questionnaire (RMDQ), after 4, 8, and 52 weeks [25]. These data provide the opportunity to perform an ancillary analysis of the guideline implementation trial and study the STarT’s predictive ability in a population consisting solely of patients consulting general practice. In a UK primary care setting, a 30% improvement between baseline and follow-up has been estimated as guidance for defining clinically relevant improvement in function when applying the RMDQ [26].

### The aim

The aim was to study whether the STarT score for patients consulting general practice with LBP was predictive of a functional improvement of 30% in the RMDQ score after 8 weeks.

## Methods

### Design and setting

This was an ancillary analysis of a cluster randomised controlled trial on guideline implementation for LBP in Danish general practice. Reporting of the present study follows the STROBE Statement [27].

From January 2013 to July 2014, 60 general practices in the North Denmark Region participated in a guideline implementation trial. The cluster randomised controlled trial compared two strategies for supporting the implementation of LBP guidelines with the primary aim of reducing the referral of patients from primary care to secondary care. General practices in the intervention group had an outreach visit from a guideline facilitator, were offered access to feedback on their treatment of low back pain, and had the opportunity to score their patients with STarT (which was embedded in their electronic medical record). The GPs’ STarT scoring results are not reported in this study. Practices participating in the guideline implementation study had a project module installed in their electronic medical record system, and GPs were encouraged to perform diagnostic coding during consultations with LBP patients [25]. The International Coding for Primary Care (ICPC-2) diagnostic codes L02, L03, L84, and L86 [28] triggered a pop-up in the medical record system. If a patient met the inclusion criteria, the GP invited the patient to participate in the guideline implementation study. The inclusion criteria were consulting general practice with LBP of any duration for the first time within 3 months, age 18 to 65 years, with or without associated radiculopathy, and a complete STarT questionnaire at baseline. The exclusion criteria were insufficient language skills to fill out questionnaires in Danish, pregnancy, and serious underlying disease (e.g., signs of fracture, osteoporosis, cauda equina syndrome, malignancy, or spinal inflammatory arthritis) [29]. Patients consenting to participate in the guideline implementation study were informed that participation with questionnaires was not a requirement for study participation, but they were encouraged to do so. For this ancillary analysis, we included patients from both the intervention group and the control group who filled in the RMDQ and had a complete STarT questionnaire at baseline. Patients with perfect function (RMDQ = 0) at baseline were excluded.

Patients filled in a questionnaire at home after the initial consultation and were sent follow-up questionnaires after four, eight, and 52 weeks. Patients could choose to complete the questionnaires on the internet or to fill out and return paper versions. Paper versions of the questionnaires were sent to the research unit in a prepaid envelope and the responses were typed into the database by two of the researchers (AR and CEJ). When completing the questionnaires on the internet, the data were directly stored in the project’s database. Every nine STarT items were programmed with a limiter, prompting the patient to respond to all nine items before access to page two of the questionnaire was possible. The 23 RDMQ items were, however, not provided with a limiter. The use of limiters to avoid missing values was not possible in the paper version of the questionnaires, but text was inserted encouraging a reply to all questions. If patients did not respond to a questionnaire, reminders (emails or postal letters) were sent following one- and two-week delays [25]. The database was hosted by an external data manager at the North Denmark Region Department of Information Technology. The project database was provided with access login, written recording and daily backup copying.

### Outcome measure

The predictor variable was the patient reported STarT risk group (low, medium, or high) at baseline. The primary outcome was assessed by a relative risk combining the low-risk group and the medium-risk group and comparing these to the high-risk group in terms of good outcomes. A good outcome was defined as receiving a minimal clinically relevant improvement in the RMDQ score (0–23 points) after 8 weeks [30]. The outcome was dichotomised using a standard cut-off at 30% improvement [21, 22, 26, 31]. As previous studies also included a secondary cut-off point between the low-risk group and the medium-risk group, this was applied as a secondary analysis. Furthermore, clinically relevant improvements after four and 52 weeks were included as secondary analyses.

### Statistical analysis

For each STarT risk group, baseline characteristics were presented with numbers (%) for categorical variables and mean (sd) or median [iqr] for continuous variables. Baseline characteristics were patients’ age, gender, college education (y/n), employment (y/n), sick leave within 14 days (hours), RMDQ score (0–23 points) [30], numerical pain rating (0–10 points) [32], and self-reported health (EQ VAS, 0–100 points) [33]. Differences in baseline characteristics were tested by Fischer’s Exact test for categorical outcomes (gender, education level, and employment status), with the Student’s t-test (age, numerical pain rating, EQ VAS, and RMDQ), or by the Mann-Whitney test (sick leave) for continuous outcomes. For continuous outcomes, the tests were only comparing the low-risk group with the high-risk group.

For estimating the predictive ability of the STarT, a combination of the low-risk group and the medium-risk group was compared with the high-risk group and the low-risk group was compared with the medium-risk group + the high-risk group by relative risks. A regression analysis was performed to study whether the allocation group in the guideline implementation study was likely to have introduced bias into the estimates. The regression analysis includes baseline RMDQ score, allocation group in the cluster randomised controlled trial together with all the following baseline variables: age (continuous), gender (male/female), college level education (yes/no), employment status (employed, yes/no), sick leave (any LBP-related sick leave 14 days prior to baseline), numerical pain rating (continuous), and EQ VAS (continuous).

The study size was 441 patients by including all patients with a complete STarT from the guideline implementation trial [25]. Single responses from the 23-item RMDQ were coded 0 (no) if they were missing, allowing the inclusion of these observations in the analysis. Patients with a RMDQ score of 0 (optimal function) were excluded from the analysis as they could not achieve a 30% improvement. Throughout the analyses, a *P* value of <0.05 was considered statistically significant. Analyses were performed using Stata, IC version 14.0 (College Station, Texas, USA).

## Results

**Fig. 1:**
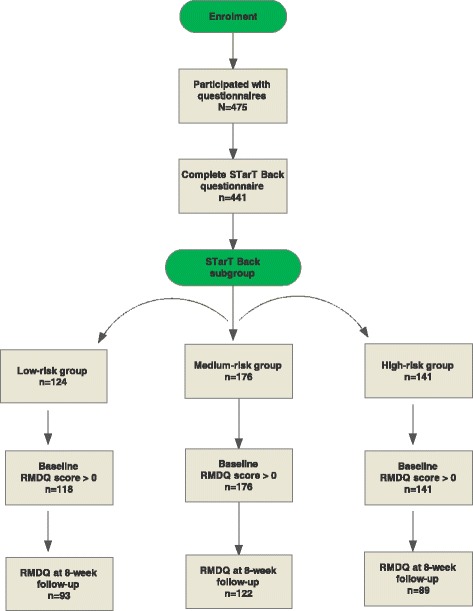
Flow chart. *Note:* 1101 patients were included in the cluster randomised trial, from which 475 participated with questionnaires. 441 patients had a complete STarT score and were included in this study.

Between January 2013 to July 2014, 1101 patients were included in the cluster randomised controlled trial. A subpopulation of 475 patients participated with questionnaires and was eligible to be included in this ancillary analysis. Among the 475 patients eligible for this analysis, 441 had a complete STarT questionnaire and formed our study population (Fig. 1). According to STarT, 124 (28%) scored low, 176 (40%) scored medium, and 141(32%) scored high (Table 1).

**Table 1:** Baseline characteristics

Patient characteristics	STarT low-risk *N* = 124	STarT medium-risk *N* = 176	STarT high-risk *N* = 141	*P*-value
Age (years)	46.8 (11.6)	45.2 (11.4)	43.7 (11.0)	0.029^*^
Gender (male)	63 (50.8%)	81 (46.0%)	66 (46.8%)	0.705^†^
College-level education (yes)	41 (33.6%)	46 (26.6%)	17 (12.4%)	< 0.001^†^
Employed (yes)	94 (76.4%)	136 (78.2%)	94 (68.6%)	0.144^†^
Sick leave with LBP, hours during 14 days	0 [0; 8]	4.5 [0; 18.5]	18 [2; 40]	< 0.001^ǂ^
RMDQ score (0–23 points)	8.7 (4.9)	14.0 (4.7)	17.3 (4.2)	< 0.001^*^
Numerical pain rating (0–10 points)	4.6 (2.1)	6.3 (2.0)	7.2 (1.7)	< 0.001^*^
EQ VAS (0–100 points)	64.4 (20.9)	57.5 (20.3)	43.7 (21.8)	< 0.001^*^

*Note*: STarT Back Tool is a patient-reported nine-item questionnaire, which subgroups patients according to risk of complexity and persistent symptoms. ^*^Comparing the low-risk group and the high-risk group and tested by Student’s T-test. ^†^Comparing all groups by Fischer’s Exact test. ^ǂ^Comparing the low-risk group and the high-risk group and tested by the Mann-Whitney test. Data are mean (sd), number (%), or median [iqr]. The numbers of missing values were as follows: education *n* = 9, employment status *n* = 7, sick leave *n* = 68, numerical pain rating *n* = 12, and EQ VAS *n* = 6

Patients eligible for this study (*n* = 475) were older than patients not eligible (*n* = 626); mean of 45.2 years vs 41.7 years. However, in terms of age and referral to secondary care, there were no statistically significant differences between patients eligible for this study and the other patients included in the guideline implementation trial. From the eligible subpopulation of 475 patients, 304 (64.0%) patients provided complete information regarding STarT at baseline and completed the RMDQ questionnaire at baseline and after 8 weeks.

After 8 weeks, 61 (65.6%) in the low-risk group, 67 (54.9%) in the medium-risk group, and 33 (37.1%) in the high-risk group achieved a 30% improvement in RMDQ. High-risk patients were at a higher risk of not achieving a 30% improvement in RMDQ after 8 weeks compared with patients in the low- and medium-risk groups (RR 1.61 [1.20–2.15, *p* < 0.001]). For all comparisons, the higher STarT group(s) were at higher risk of not achieving a clinically relevant improvement in RMDQ compared with other patients (the low-risk group + the medium-risk group (Table 2).

**Table 2:** STarT Back Tool risk groups and 30% improvement in the Roland Morris Disability score

STarT Back cut-off	Low vs Medium/High			Low/Medium vs High		
Follow-up	RR	95% CI	*P*-value	RR	95% CI	*P*-value
4 weeks	1.64	(1.26–2.15)	< 0.001	1.71	(1.18–2.49)	0.002
8 weeks	1.38	(1.13–1.70)	0.003	1.61	(1.20–2.15)	< 0.001
52 weeks	1.21	(1.02–1.43)	0.040	1.36	(1.08–1.72)	0.003

*Note:* Relative risk (RR) of not achieving a clinically relevant improvement in function (30% improvement in the Roland Morris Disability score). The higher RR comparing Low/Medium vs High is equivalent to more desirable functional outcomes for the ‘Low/Medium group’

A regression analysis to study the effect of patients’ allocation group in the guideline implementation trial showed no statistically significant or clinically relevant changes in estimates (Table 3). The only factors staying significantly predictive of functional improvement in the adjusted model were the STarT group and EQ VAS.

**Table 3:** Odds of STarT Back Tool high-risk patients achieving a 30% improvement in the Roland Morris Disability score after 8 weeks

Possible risk factors	Crude OR	95% CI	*P*-value	Adj OR^a^	95% CI	*P*-value
STarT Back Tool (high-risk group)^b^	0.40	0.24–0.67	< 0.001	0.36	0.18–0.73	0.004
Allocation group in the cluster randomized trial (intervention group)	0.99	0.63–1.55	0.963	0.95	0.55–1.62	0.840
Age (high)	1.00	0.98–1.02	0.995	1.00	0.97–1.02	0.790
Gender (male)	0.95	0.60–1.50	0.833	0.86	0.50–1.48	0.574
College education (yes)	1.12	0.66–1.90	0.678	0.71	0.38–1.32	0.278
Employment status (yes)	1.71	1.01–2.90	0.046	1.56	0.73–3.37	0.253
Sick leave (yes)^c^	1.27	0.78–2.08	0.335	1.45	0.79–2.65	0.232
Numerical pain rating (high)	0.95	0.85–1.06	0.341	1.05	0.88–1.25	0.565
EQ VAS (high rated health)	1.02	1.01–1.03	0.001	1.02	1.01–1.04	0.006
Baseline RMDQ score	0.99	0.95–1.03	0.600	1.05	0.98–1.13	0.173

*Note:* Crude odds ratios (Crude OR) and adjusted odds ratios.^a^Adjusted odds ratio (Adj OR) from a multiple regression analysis including all tabulated variables.^b^ Comparing the low-risk and medium-risk groups with the high-risk group. Age, numerical pain rating, and EQ VAS are included as continuous variables. STarT Back Tool, allocation in the previous study, gender, college education, employment status, and sick leave are included as dichotomised variables.^c^ Sick leave was coded yes if the patient reported any amount of LBP-related sick leave 14 days prior to baseline

## Discussion

In patients with LBP consulting Danish general practice, the STarT subgroups were predictive of the patients’ functional improvement measured by the RMDQ score. After 8 weeks, 61 (65.6%) in the low-risk group, 67 (54.9%) in the medium-risk group, and 33 (37.1%) in the high-risk group achieved a 30% improvement in the RMDQ score. High-risk patients were at a 61% higher risk of not achieving a 30% improvement in the RMDQ score after 8 weeks compared with the combined group of patients at medium risk and patients at low risk according to STarT.

In previous studies, follow-up has been applied after 12 weeks [21, 22]; therefore, the follow-up point after 8 weeks, being the closest to the main trial, was applied as the primary analysis in this study and this deviation from previous studies can be considered a limitation. However, the use of follow-up points after 4 weeks (short term), 8 weeks (medium term), and 52 weeks (long term) is considered a strength of this study. Furthermore, neither the choice of follow-up period nor the choice of cut-off used to dichotomize the STarT score significantly changed the conclusion. This similarly strengthens the interpretation of results. This is an ancillary analysis of data collected for a cluster randomised controlled trial, where general practices and their patients were randomised to different strategies to manage LBP. This may weaken the interpretation of results. In particular, the integration of the STarT in general practitioners’ medical record systems in the intervention group could have biased the results. Applying STarT to guide treatment has been found to be effective in improving patients’ RMDQ scores [15], and this improvement has been found to be particularly present among high-risk patients [15]. Thus, offering GPs the opportunity to use STarT might have led to an underestimation of the RRs in this study. However, including the allocation group in an adjusted model did not affect the results. Patients were given the questionnaire at the consultation at the day of inclusion. About 90% replied the same day; however, a few patients replied with a one- or two-day delay. This delay might have improved patients’ RMDQ score at baseline and might have caused an underestimation of the real improvement in the RMDQ score. In our study, this could lead to a small underestimation of the relative risks. It could have been of interest to adjust for the duration of LBP or even to exclude patients with pain lasting less than 14 days, as STarT has been found to be unable to predict outcome among these patients [23]. These data were, however, not available in the present study.

In a study from the US, patients were recruited directly from physiotherapy clinics, where the STarT could identify distinctive patterns between the low-risk group and the high-risk group but not when comparing the medium-risk group with the other two groups [17]. In line with the present study, the STarT’s ability to identify patients at risk of higher levels of disability by the Oswestry Disability Index has been supported in a study recruiting from a university community in Canada. They recruited participants by advertising in a local newspaper to screen for LBP in a chiropractic clinic [18]. In contrast to these findings, STarT has not been able to predict outcomes in two studies of patients seeking care at chiropractic clinics in Denmark using the RMDQ score as an outcome measure and the UK using the Patient Global Impression of Change as an outcome measure [18, 20]. A Danish study with a combined population from physiotherapy clinics and general practices found the STarT was able to predict improvements in the RMDQ score (RR 2.4 for low-risk vs. medium-risk and RR 2.8 for low-risk vs. high-risk) [21]. Lower predictive ability has been found in Danish secondary care (RR 1.5 for low-risk vs. medium-risk and RR 1.7 for low-risk vs. high-risk) [22]. The STarT was originally validated in a UK general practice setting [34] and in line with the original STarT trial, the present study consists exclusively of patients enrolled when consulting their general practitioner for LBP, which may increase generalizability to other general practice settings.

Compared with the present study, previous studies had very similar baseline characteristics in terms of pain rating [17–23]. In addition to the healthcare setting, pain duration seems important when comparing STarT subgroups. STarT has not been found suitable for patients with acute pain, especially not for patients with pain for less than 2 weeks [23, 24].

Findings from this study confirm the results from the original trial validating the STarT [34], thereby adding knowledge to support the ability of STarT to predict improvements in the RMDQ score in general practice settings. Even though STarT is found to be predictive of functional improvements in this study, this does, however, not support the effectiveness of the targeted treatment arms, which were applied in UK studies. Therefore, more research on the effect of stratifying treatment according to the STarT outside of the UK is needed.

## Conclusion

The STarT subgroups were predictive of functional improvement in Danish general practice. This study supports wider implementation of the STarT.

## Abbreviations

EQ VAS: EuroQol Visual Analogue Scale
GP: General Practitioner
LBP: Low Back Pain
NICE: The National Institute for Health and Care Excellence
RMDQ: Roland Morris Disability Questionnaire
RR: Relative Risk
STarT: STarT Back Tool
UK: United Kingdom
